# Supplementary material for: Quantitative interactome of a membrane Bcl-2 network identifies a hierarchy of complexes for apoptosis regulation
Source: Nat Commun. 2017 Jul 13;8:73. doi: 10.1038/s41467-017-00086-6 (PMC5509671; doi:10.1038/s41467-017-00086-6)
Supplement: Supplementary file 1 — Supplementary Information [file 41467_2017_86_MOESM1_ESM.pdf]

SI GUIDE

Title: Supplementary Information
Description: Supplementary Figures, Supplementary Tables, Supplementary Methods, and Supplementary References

Title: Peer Review File
Description:

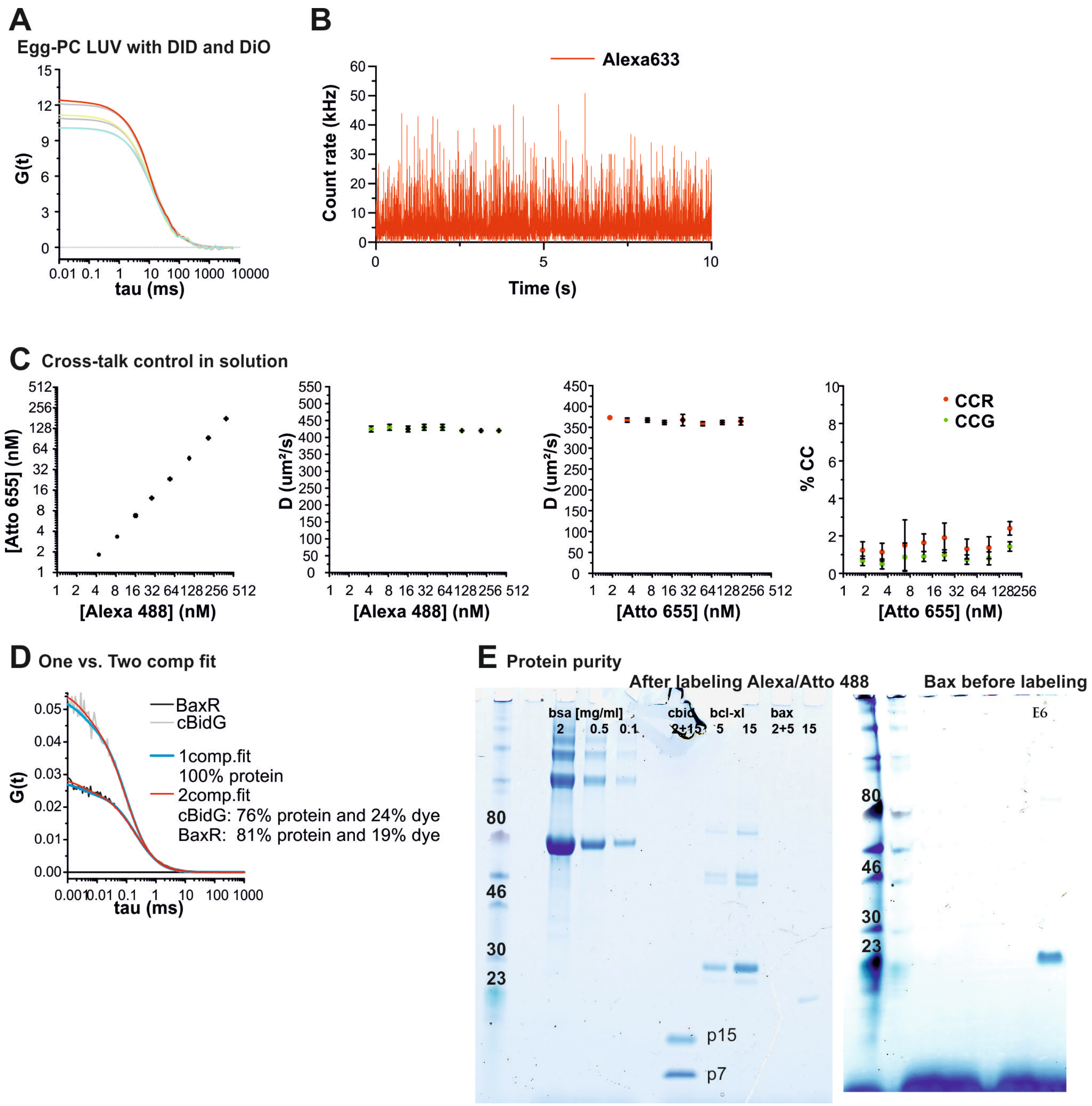

Supplementary Figure 1: Control measurements. A) Exemplary AC and CC curves on egg PC LUVs containing >0.05% DiO and DiD. B) Exemplary fluorescence intensity trace on free Alexa633. C) Cross talk control using free Alexa488 and Atto655 mixed at different concentration. Shown is one (of n=3) exemplary experiment with mean and error bars as s.d. based on three technical repetitions. From n=3 independent experiments a %CC of μ:1.4, σ: 0.7 was calculated. The different graphs plot the protein concentration, the D of both molecules and the %CC. D) Exemplary 1 component and 2 component fittings of the AC curves of Bax_R and $cBid_G$ in solution. E) SDS-PAGE of $cBid_G$, $Bcl\text{-}xL_G$ and Bax_G and Bax before the labeling process as a visual guide for protein purity. More information in the Supplemental Methods.

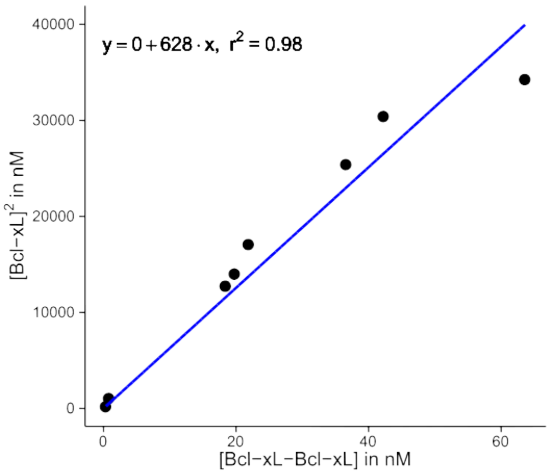

Supplementary Figure 2: Bcl-xL homodimerization as measured by FCS. Theoretical K_D value of 628 nM calculated when assuming equilibrium conditions of Bcl-xL homodimerization of two differently labeled Bcl-xL proteins. Plotted is the squared concentration of free Bcl-xL proteins against the sum of all possible homodimeric Bcl-xL species (combinations of unlabeled and labeled proteins). Please note that the complex concentrations calculated from the %CC were corrected for the degree of labeling of the proteins and a Mendel-based distribution of red-red, green-green and red-green complexes, but not for partial overlap of the detection volumes. Data are from n=4 experiments.

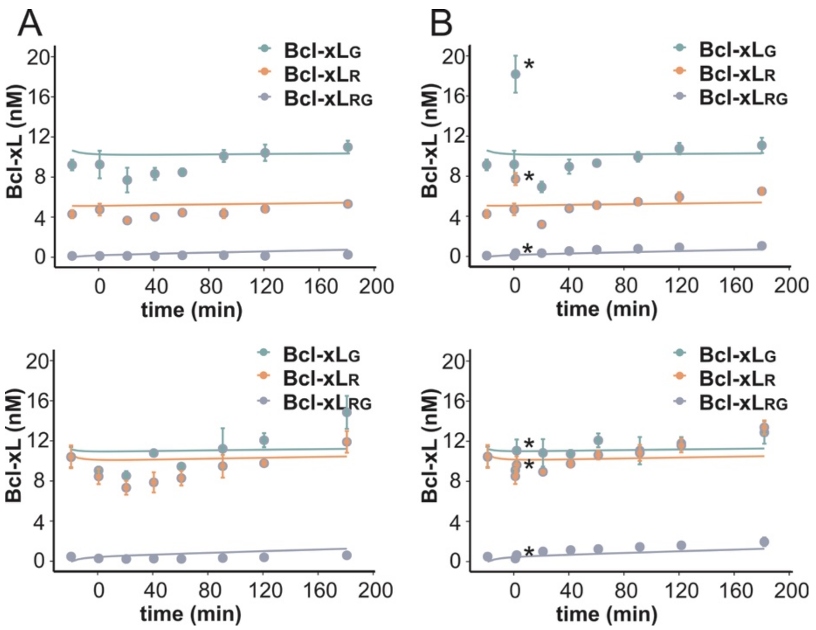

Supplementary Figure 3: Best fit of ODE model in scenario 1 to the kinetics of Bcl-xL dimerization measured by FCCS in Figure 3B.

(A) Fitting to the control data after buffer addition and (B) fitting to the data after cBid addition. The upper and the lower panels show two independent experiments with mean and error bars as s.d. of three technical repetitions. To do the ODE fitting in B, we took the t=0 data point from A (without cBid). This is applicable as A and B present data of one sample split into two equal parts directly before measuring t=0. The first data point in presence of cBid is introduced as t=1min and highlighted in the figure by *. This is applicable as each data point had a total FCS measurement time of 2 min. The model corresponding to scenario 1 cannot qualitatively reproduce the occurrence of Bcl-xL$_G$-Bcl-xL$_R$ dimer particles after addition of cBid. Instead, cross-correlating particles appear in same amounts irrespective of cBid addition.

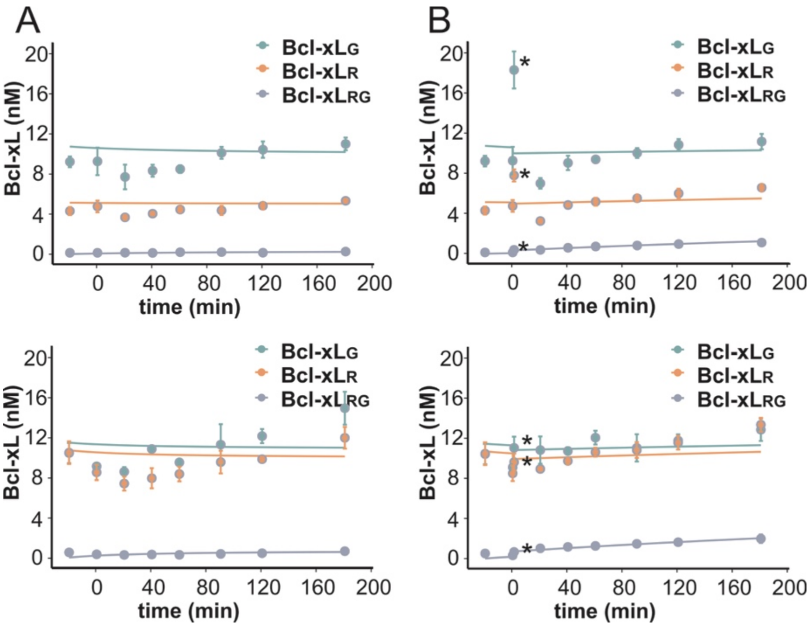

Supplementary Figure 4: Best fit of ODE model in scenario 3 to the kinetics of Bcl-xL dimerization measured by FCCS in Figure 3B.

(A) Fitting to the control data after buffer addition and (B) fitting to the data after cBid addition. The upper and the lower panel show two independent experiments with mean and error bars as s.d. of three technical repetitions. To do the ODE fitting in B, we took the t=0 data point from A (without cBid). This is applicable as A and B present data of one sample split into two equal parts directly before measuring t=0. The first data point in presence of cBid is introduced as t=1min and highlighted in the figure by *. This is applicable as each data point had a total FCS measurement time of 2 min.

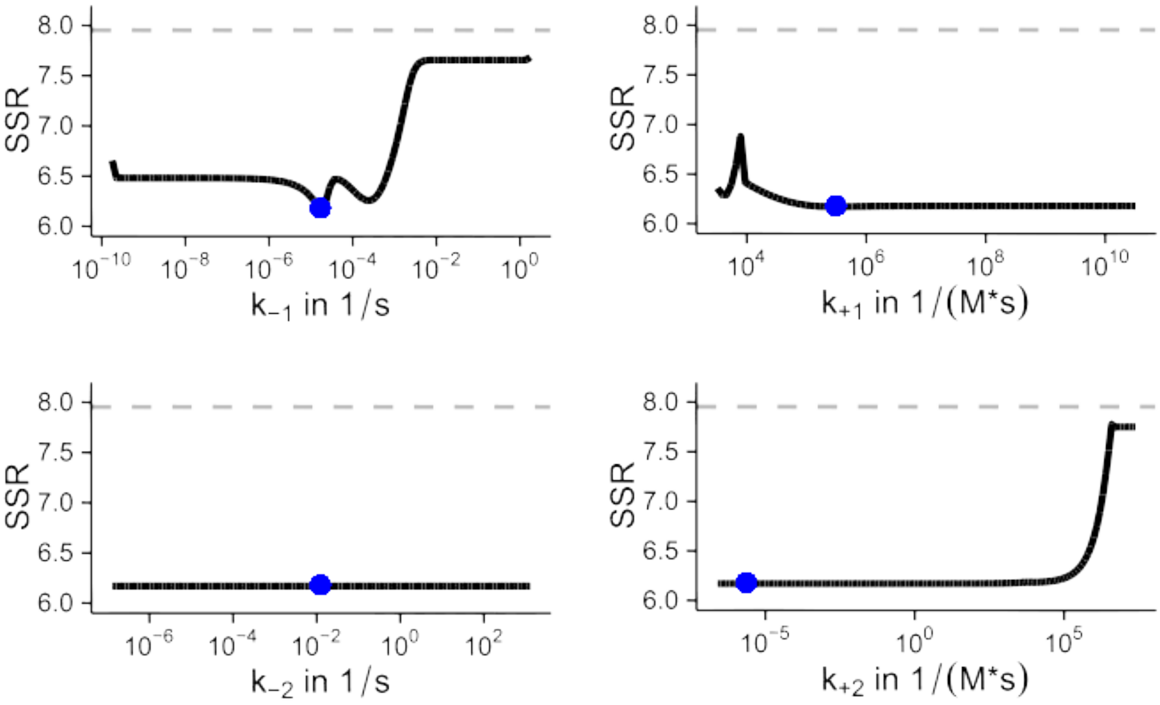

Supplementary Figure 5: Likelihood profiles of parameters defining rate constants of model of interaction scenario 1. The fitted parameter is shown as blue dot, the re-optimized SSR is shown as black line and the dashed grey line indicates the confidence limit.

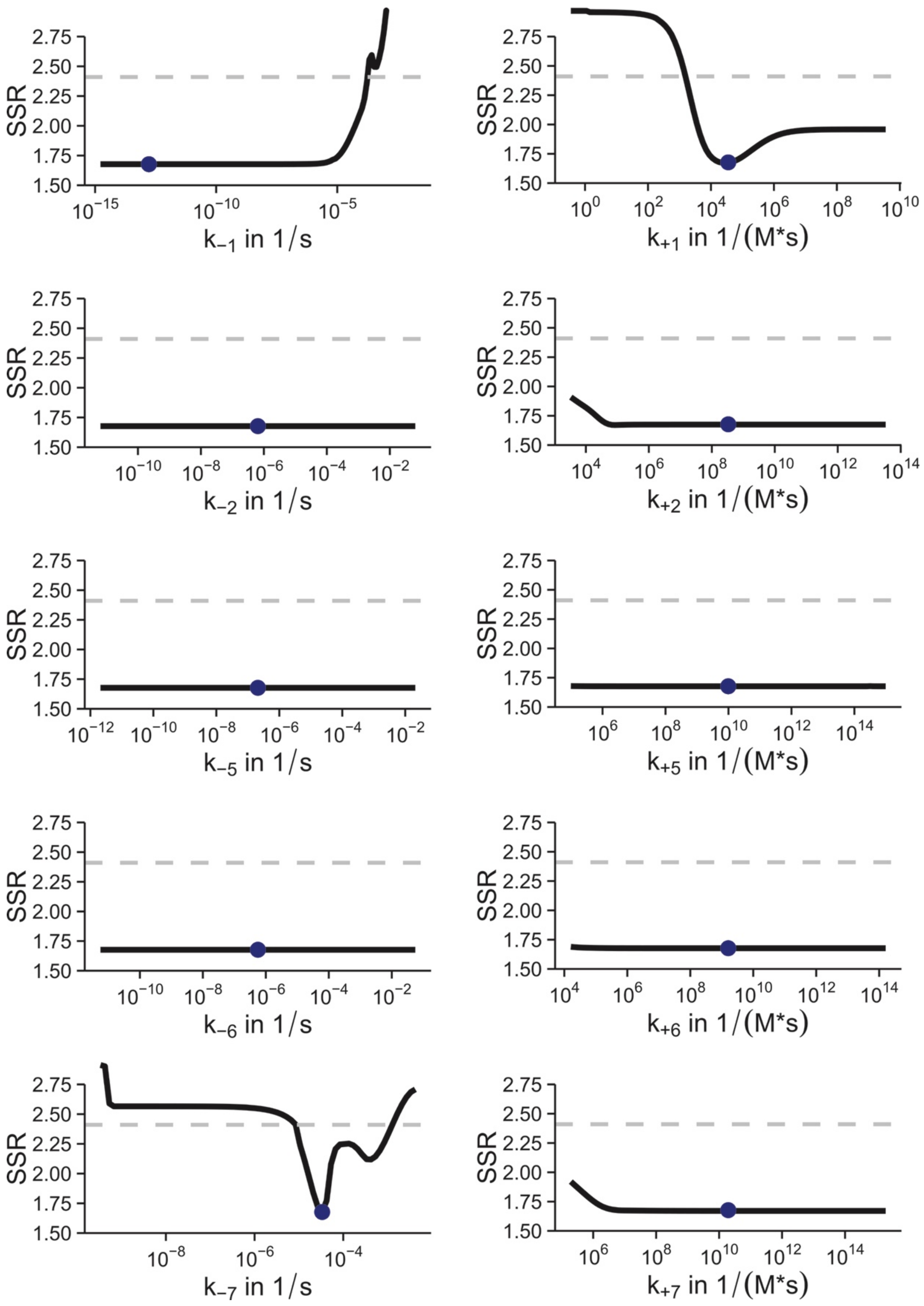

Supplementary Figure 6: Likelihood profiles of parameters defining rate constants of model of interaction scenario 3. The fitted parameter is shown as blue dot, the re-optimized SSR is shown as black line and the dashed grey line indicates the confidence limit.

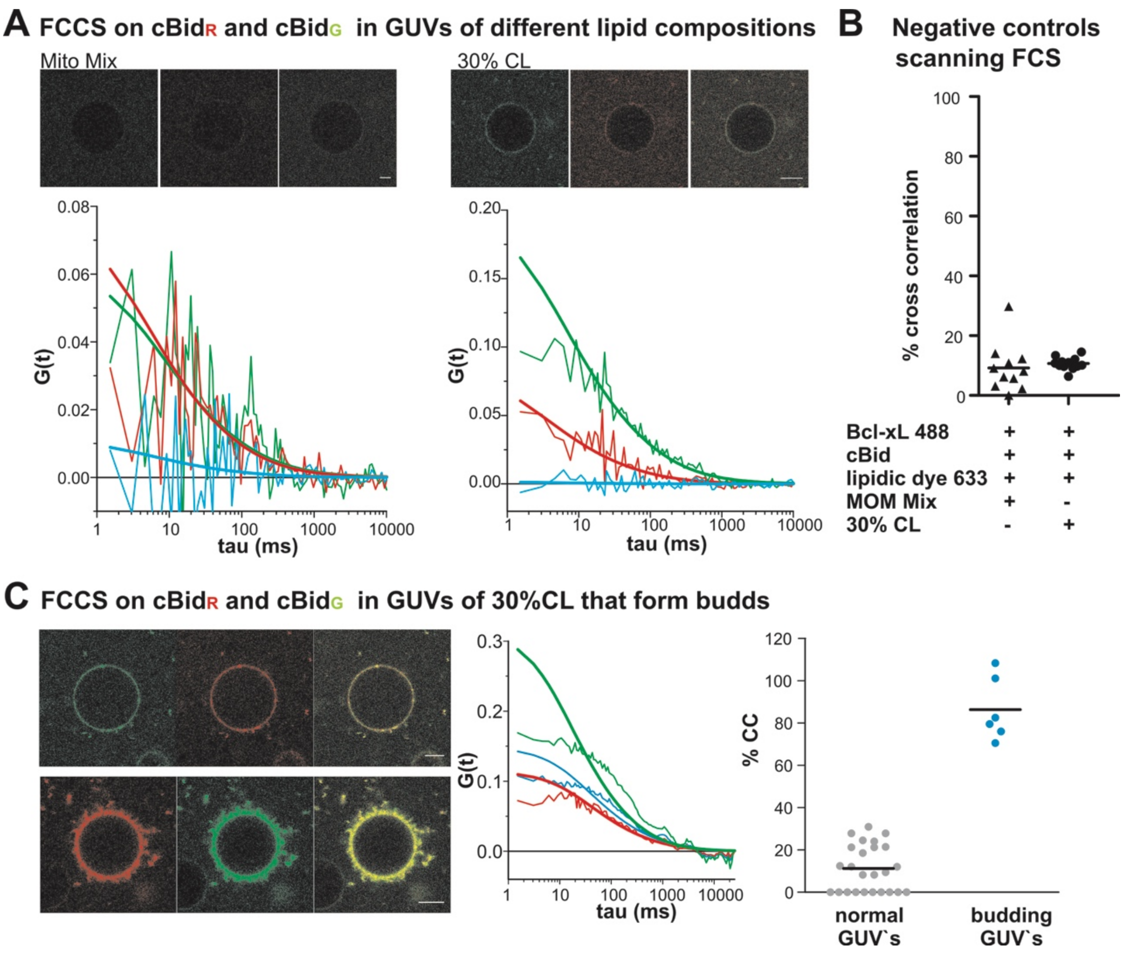

Supplementary Figure 7: Different test experiments in membranes. A) AC- and CC curves of cBid molecules in GUVs using the lipid mixture mimicking the MOM or the 30% CL lipid mix. B) Negative controls for cross-correlation in membranes. As negative control the %CC between Bcl-xL$_G$ and DiD, two molecules not interacting was measured in both used lipid mixtures (n=3). C) Cross-correlation between cBid molecules in GUVs formed by 30% CL (n=4). Here, two different situations could be detected. GUVs with a smooth surface show a very low cross-correlation similar to the negative control (left panel upper GUV and right panel "normal GUVs". In contrast GUVs that show budding (left panel, lower GUV) showed high level of cross-correlation (right panel "budding GUVs"). Moreover, the AC and CC curves of budding GUVs showed a slow diffusion that could not be fitted properly to a 2D diffusion (middle panel). We assume that the curves describe the movements on buds on the GUV surface rather than single molecule diffusion in the membranes and as each bud contain likely several cBid molecules we detect a high %CC. Scale bar 10 µm.

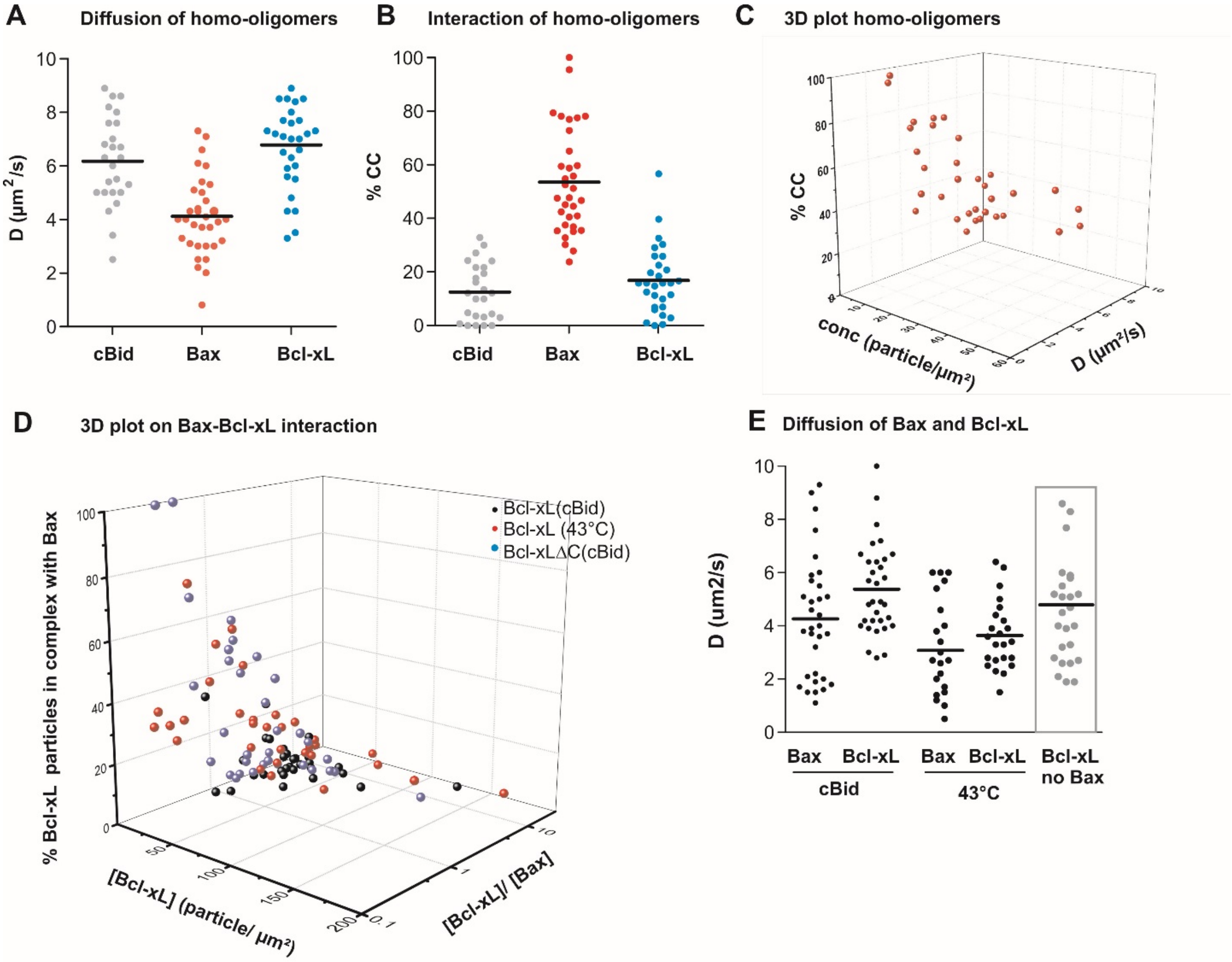

Supplementary Figure 8: Additional information on Bax homo- and hetero-oligomer formation in membranes.

(A-B) Diffusion coefficients (A) and cross-correlation (B) of membrane embedded cBid and Bax and Bcl-xL homo-oligomers using GUVs of the 30% CL mix (cBid n=4; Bax n=5; Bcl-xL n=5). The CC values from 3 of 5 experiments studying Bax and Bcl-xL homo- interactions used here were already published in[1]. C) 3D graph of Bax-homo-oligomers in the membranes of individual GUVs plotting the relationship between concentration, diffusion and %CC (n=5). D) Related to Figure 6E. 3D graph plotting the relationship between Bax_R and Bcl-xL_G concentration and percentage of Bcl-xL molecules in hetero-complexes with Bax. Data points from GUVs in which protein insertion was triggered by cBid are black dots; when heat was used red dots and when cBid and Bcl-xlΔCT were used blue dots. E) Related to Figure 6E. Diffusion coefficients of membrane embedded Bax_G and Bcl-xL_R using GUVs of the 30% CL mix. Protein membrane-insertion was activated by cBid or heat. In data in the box show the diffusion coefficients of Bcl-xL in presence of only cBid taken from Figure 5A for comparison.

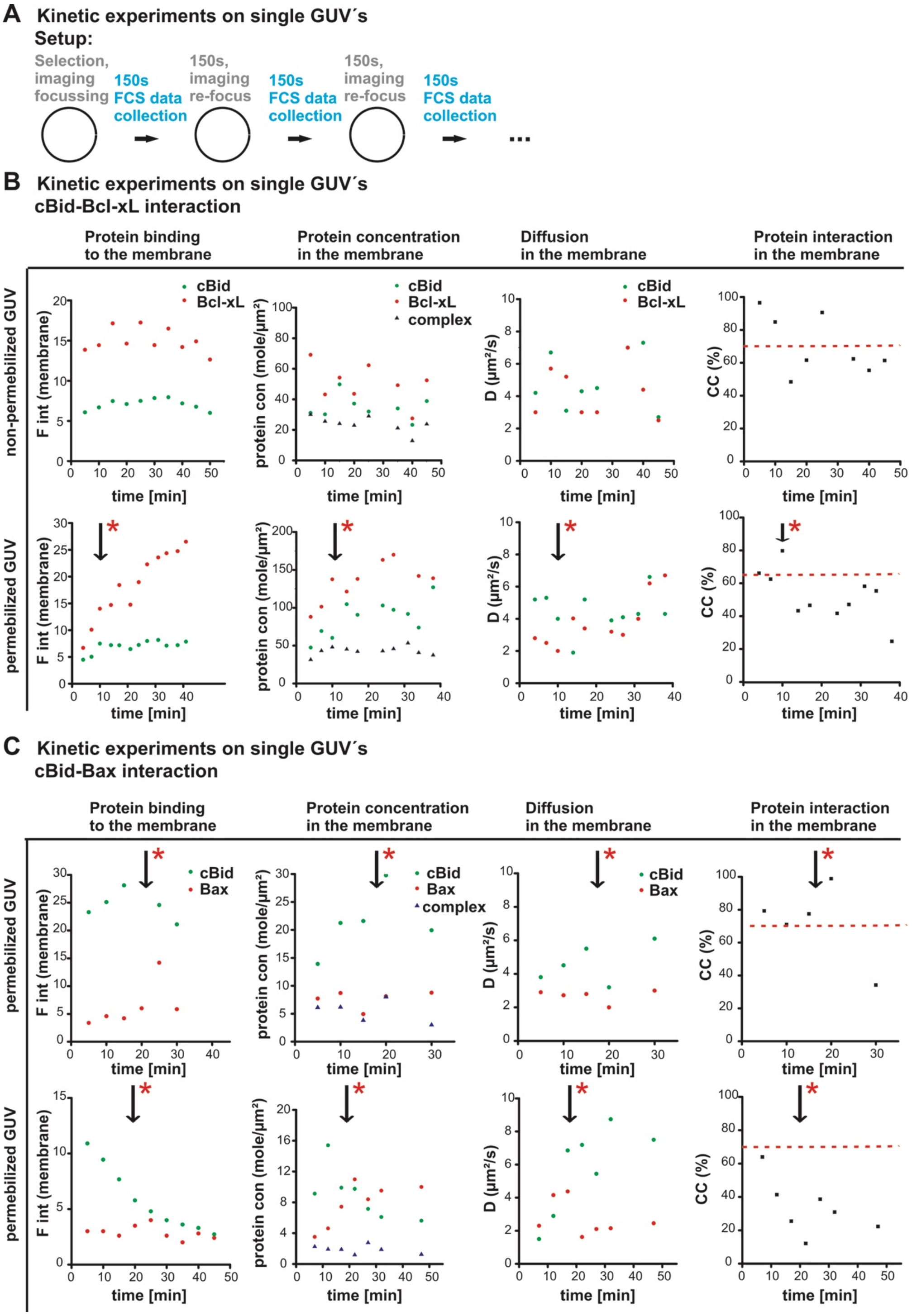

Supplementary Figure 9: Kinetic experiments of Bcl-2 protein interactions in membranes. A) Schematic drawing on how the experiment was performed. (B-C) Kinetic experiments of the interaction of $cBid_G$ with $Bcl\text{-}xL_R$ (B) or Bax_R (C). The first panel on the left shows changes in the fluorescence intensity of membrane embedded $cBid_G$, $Bcl\text{-}xL_R$ or Bax_R over time measured by imaging. The second panel shows changes in the protein and complex concentration over time measured by FCS. Concentration and fluorescence intensity are stable or rising except for $cBid_G$ in presence of Bax, where cBid molecules seem to be released from the membrane. The third panel follows D over time. For cBid-Bcl-xL D is relatively stable over time, while the D of cBid in presence of Bax can rise in line with cBid release from the complex. The right panel shows changes in the cross-correlation over time. The red line indicates the maximal possible cross-correlation and the arrow indicates the time point when the GUV was permeabilized.

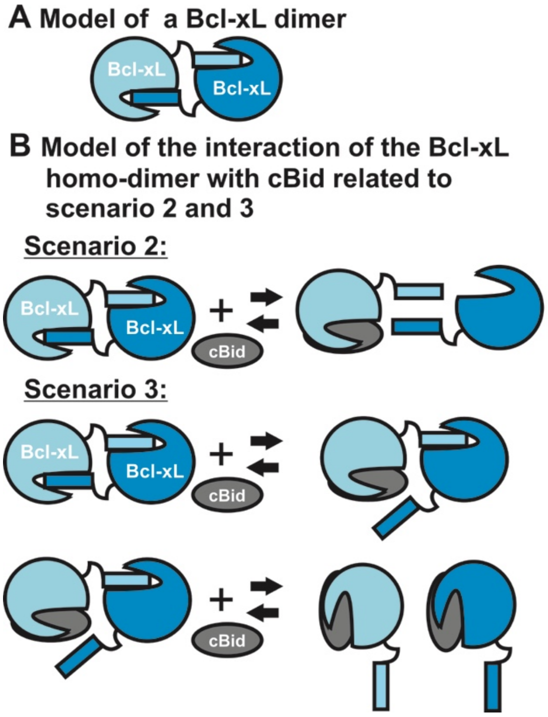

Supplementary Figure 10: Model of Bcl-xL dimerization. A) Schematic drawing of a Bcl-xL homo-dimer. B) Schematic models of the interaction of cBid with a Bcl-xL homo-dimer related to Scenarios 2 and 3.

Name	Function
Auto-correlation function	$$G(\tau) = \frac{\langle \delta F(t) \delta F(t+\tau) \rangle}{\langle F(t) \rangle^2}$$
Cross-correlation function	$$G_x(\tau) = \frac{\langle \delta F_R(t)\, \delta F_G(t+\tau) \rangle}{\langle F_R(t) \rangle \langle F_G(t) \rangle}$$
Fraction of molecules in two color complex	$C_{RG} = \frac{G_{0,x}}{G_{0,G}}$ or $C_{RG} = \frac{G_{0,x}}{G_{0,R}}$
Diffusion coefficient	$$D = \frac{\omega_0^2 4t_D}{4t_D}$$
3D diffusion for solution measurements	$$G_{3D}(\tau) = \frac{1}{N}\left(1 + \frac{\tau}{\tau_D}\right)^{-1} \frac{1}{\sqrt{1 + \frac{\tau}{S^2 \tau_D}}}$$
Dual-focus scanning FCS for measurements in membranes	$$G_{12}(\tau) = \frac{1}{C\pi s\omega_0}\left(1 + \frac{4D\tau}{\omega_0^2}\right)^{-1/2}\left(1 + \frac{4D\tau}{\omega_0^2 s^2}\right)^{-1/2} \exp\left(-\frac{d^2}{\omega_0^2 + 4D\tau}\right)$$

Supplementary Table 1: Functions used for the FCS analysis. *G* is the autocorrelation and G_x the cross-correlation function; τ the lag time for correlation; *F*(*t*) the fluorescence intensity as a function of time (*t*); ⟨ ⟩ corresponds to time averaging ($\delta F(t) = F(t) - \langle F(t) \rangle$); F_R and F_G are the fluorescence intensity in the red and green channels; C_{RG} is the concentration molecules in the two color complex; $C_{RG,t}$ the concentration of all particles having both colors; $G_{0,x}$ is the cross-correlation amplitude; $G_{0,R}$ ($G_{0,G}$) is the amplitude or the red (or green) autocorrelation curve; *D* is the diffusion coefficient; *N* is the average number of detected molecules; t_D is the average time the fluorophore stays in the focal volume; G_{12} is the spatial cross-correlation curve comparing the two foci; and *d* is the distance between the two foci.

Scenario	SSR	k	n	AIC_c	Δ_i	w_i
1	6.17037	12	90	215.83	121.93	3.3e-27
**2**	**1.4318**	**14**	**90**	**93.90**	**0**	**1.0**
3	1.67701	18	90	128.16	34.26	3.6e-08

Supplementary Table 2: SSR, AIC values and differences in AIC values of models of different interaction scenarios. *k* denote the number of fitted parameters, *n* the number of experimental data points the model was fitted to. AIC_c denotes the corrected Akaike information criterion as described in Materials and Methods.

	Scenario 1	Scenario 2	Scenario 3
d([C])<br>dt	− (k2on · [C] · [B0])<br>+ (k2off · [CB0])<br>- (k2on · [C] · [B1])<br>+ (k2off · [CB1])<br>- (k2on · [C] · [B2])<br>+ (k2off · [CB2])	- (k2on · [C] · [B1])<br>+(k2off · [CB1])<br>- (k2on · [C] · [B2])<br>+(k2off · [CB2])<br>-(k3on · [B1B1] · [C])<br>+(k3off · [CB1] · [B1])<br>-(k3on · [B2B2] · [C])<br>+(k3off · [CB2] · [B2])<br>-(k3on · [B1B2] · [C])<br>+(k3off · [CB1] · [B2])<br>-(k3on · [B1B2] · [C])<br>+(k3off · [CB2] · [B1])<br>-(k2on · [C] · [B0])<br>+(k2off · [CB0])<br>- (k3on · [B1B0] · [C])<br>+(k3off · [CB1] · [B0])<br>-(k3on · [B1B0] · [C])<br>+(k3off · [CB0] · [B1])<br>-(k3on · [B2B0] · [C])<br>+(k3off · [CB2] · [B0])<br>-(k3on · [B2B0] · [C])<br>+(k3off · [CB0] · [B2])<br>-(k3on · [B0B0] · [C])<br>+(k3off · [CB0] · [B0])	+(k5off · [CB2B2])<br>-(k5on · [B2B2] · [C])<br>+(k5off · [CB1B1])<br>-(k5on · [B1B1] · [C])<br>+(k5off · [CB0B0])<br>-(k5on · [B0B0] · [C])<br>-(k5on · [B1B2] · [C])<br>+(k5off · [CB1B2])<br>-(k5on · [B1B0] · [C])<br>+(k5off · [CB1B0])<br>-(k5on · [B2B0] · [C])<br>+(k5off · [CB2B0])<br>+(k2off · [CB0])<br>-(k2on · [C] · [B0])<br>+(k2off · [CB1])<br>-(k2on · [C] · [B1])<br>+(k2off · [CB2])<br>-(k2on · [C] · [B2])<br>-(k6on · [CB0B0] · [C])<br>+(k6off · [CB0B0C])<br>-(k6on · [CB1B1] · [C])<br>+(k6off · [CB1B1C])<br>-(k6on · [CB2B2] · [C])<br>+(k6off · [CB2B2C])<br>-(k6on · [CB1B2] · [C])<br>+(k6off · [CB1B2C])<br>-(k6on · [CB1B0] · [C])<br>+(k6off · [CB1B0C])<br>-(k6on · [CB2B0] · [C])<br>+(k6off · [CB2B0C])
d([B1])<br>dt	-(k1on · [B1] · [B2])<br>+(k1off · [B1B2])<br>-(k1on · [B1] · [B0])<br>+(k1off · [B1B0])<br>-(k2on · [C] · [B1])<br>+(k2off · [CB1])<br>-2 ·(k1on · [B1] · [B1])<br>+ 2 ·(k1off · [B1B1])	-(k1on · [B1] · [B2])<br>+(k1off · [B1B2])<br>-(k2on · [C] · [B1])<br>+(k2off · [CB1])<br>-2 ·(k1on · [B1] · [B1])<br>+ 2 ·(k1off · [B1B1])<br>+(k3on · [B1B1] · [C])<br>-(k3off · [CB1] · [B1])<br>+(k3on · [B1B2] · [C])<br>-(k3off · [CB2] · [B1])<br>-(k1on · [B1] · [B0])<br>+(k1off · [B1B0])<br>+(k3on · [B1B0] · [C])<br>-(k3off · [CB0] · [B1])	+ 2 ·(k1off · [B1B1])<br>-2 ·(k1on · [B1] · [B1])<br>+(k1off · [B1B0])<br>-(k1on · [B1] · [B0])<br>+(k1off · [B1B2])<br>-(k1on · [B1] · [B2])<br>+(k2off · [CB1])<br>-(k2on · [C] · [B1])
d([B2])<br>dt	-(k1on · [B1] · [B2])<br>+(k1off · [B1B2])<br>-(k1on · [B2] · [B0])<br>+(k1off · [B2B0])<br>-(k2on · [C] · [B2])<br>+(k2off · [CB2])<br>-2 ·(k1on · [B2] · [B2])<br>+ 2 ·(k1off · [B2B2])	-(k1on · [B1] · [B2])<br>+(k1off · [B1B2])<br>-(k2on · [C] · [B2])<br>+(k2off · [CB2])<br>-2 ·(k1on · [B2] · [B2])<br>+ 2 ·(k1off · [B2B2])<br>+(k3on · [B2B2] · [C])<br>-(k3off · [CB2] · [B2])<br>+(k3on · [B1B2] · [C])<br>-(k3off · [CB1] · [B1])<br>-(k1on · [B1] · [B0])<br>+(k1off · [B2B0])<br>+(k3on · [B2B0] · [C])<br>-(k3off · [CB0] · [B2])	+(k1off · [B2B0])<br>-(k1on · [B2] · [B0])<br>+(k1off · [B1B2])<br>-(k1on · [B1] · [B2])<br>+2 · (k1off · [B2B2])<br>-2 · (k1on · [B2] · [B2])<br>+(k2off · [CB2])<br>-(k2on · [C] · [B2])
d([B0])<br>dt	-(k1on · [B1] · [B0])<br>+(k1off · [B1B0])<br>-(k1on · [B2] · [B0])<br>+(k1off · [B2B0])<br>-2 ·(k1on · [B0] · [B0])<br>+ 2 ·(k1off · [B0B0])<br>-(k2on · [C] · [B0])<br>+(k2off · [CB0])	-(k1on · [B1] · [B0])<br>+(k1off · [B1B0])<br>-(k1on · [B2] · [B0])<br>+(k1off · [B2B0])<br>-2 ·(k1on · [B0] · [B0])<br>+ 2 ·(k1off · [B0B0])<br>-(k2on · [C] · [B0])<br>+(k2off · [CB0])<br>+(k3on · [B1B2] · [C])<br>-(k3off · [CB1] · [B0])<br>+(k3on · [B2B2] · [C])<br>-(k3off · [CB2] · [B0])<br>+(k3on · [B0B0] · [C])<br>-(k3off · [CB0] · [B0])	+(k1off · [B2B0])<br>-(k1on · [B2] · [B0])<br>+(k1off · [B1B0])<br>-(k1on · [B1] · [B0])<br>+(k2off · [CB0])<br>-(k2on · [C] · [B0])<br>+2 · (k1off · [B0B0])<br>-2 · (k1on · [B0] · [B])

d([CB1)<br>dt	+(k2on · [C] · [B1])<br>-(k2off · [CB1])	+(k2on · [C] · [B1])<br>-(k2off · [CB1])<br>+(k3on · [B1B1] · [C])<br>-(k3off · [CB1] · [B1])<br>+(k3on · [B1B2] · [C])<br>-(k3off · [CB1] · [B2])<br>+(k3on · [B1B0] · [C])<br>-(k3off · [CB1] · [B0])	+ 2 · (k7off · [CB1B1C])<br>− 2 · (k7on · [CB1] · [CB1])<br>+ (k7off · [CB1B2C])<br>− (k7on · [CB1] · [CB2])<br>+ (k7off · [CB1B0C])<br>− (k7on · [CB1] · [CB0])<br>− (k2off · [CB1])<br>+ (k2on · [C] · [B1])
d([CB2)<br>dt	+(k2on · [C] · [B2])<br>-(k2off · [CB2])	+(k2on · [C] · [B2])<br>-(k2off · [CB2])<br>+(k3on · [B1B2] · [C])<br>-(k3off · [CB2] · [B2])<br>+(k3on · [B1B2] · [C])<br>-(k3off · [CB2] · [B1])<br>+(k3on · [B2B0] · [C])<br>-(k3off · [CB2] · [B0])	+ 2 · (k7off · [CB2B2C])<br>− 2 · (k7on · [CB2] · [CB2])<br>+ (k7off · [CB1B2C])<br>− (k7on · [CB1] · [CB2])<br>+ (k7off · [CB2B0C])<br>− (k7on · [CB2] · [CB0])<br>− (k2off · [CB2])<br>+ (k2on · [C] · [B2)
d([CB0)<br>dt	+(k2on · [C] · [B0])<br>-(k2off · [CB0])	+(k2on · [C] · [B0])<br>-(k2off · [CB0])<br>+(k3on · [B1B0] · [C])<br>-(k3off · [CB0] · [B2])<br>+(k3on · [B1B0] · [C])<br>-(k3off · [CB0] · [B2])<br>+(k3on · [B0B0] · [C])<br>-(k3off · [CB0] · [B0])	+ 2 · (k7off · [CB0B0C])<br>− 2 · (k7on · [CB0] · [CB0])<br>+ (k7off · [CB1B0C])<br>− (k7on · [CB1] · [CB0])<br>+ (k7off · [CB2B0C])<br>− (k7on · [CB2] · [CB0])<br>− (k2off · [CB0])<br>+ (k2on · [C] · [B0)
d([B1B1)<br>dt	+(k1on · [B1] · [B1])<br>-(k1off · [B1B1])	+(k1on · [B1] · [B1])<br>-(k1off · [B1B1])<br>- (k3on · [B1B1] · [C])<br>+(k3off · [CB1] · [B1])	-(k1off · [B1B1])<br>+(k1on · [B1] · [B1])<br>+(k5off · [CB1B1])<br>- (k5on · [B1B1] · [C])
d([B2B2)<br>dt	+(k1on · [B2] · [B2])<br>-(k1off · [B2B2])	+(k1on · [B2] · [B2])<br>-(k1off · [B2B2])<br>- (k3on · [B2B2] · [C])<br>+(k3off · [CB2] · [B2])	+(k5off · [CB2B2])<br>- (k5on · [B2B2] · [C])<br>-(k1off · [B2B2])<br>+(k1on · [B2] · [B2])
d([B1B0)<br>dt	+(k1on · [B1] · [B0])<br>-(k1off · [B1B0])	+(k1on · [B1] · [B0])<br>-(k1off · [B1B0])<br>- (k3on · [B1B0] · [C])<br>+(k3off · [CB1] · [B0])<br>- (k3on · [B1B0] · [C])<br>+(k3off · [CB0] · [B1])	-(k1off · [B1B0])<br>+(k1on · [B1] · [B0])<br>+(k5off · [CB1B0])<br>- (k5on · [B1B0] · [C])
d([B2B0)<br>dt	+(k1on · [B2] · [B0])<br>-(k1off · [B2B0])	+(k1on · [B2] · [B0])<br>-(k1off · [B2B0])<br>- (k3on · [B2B0] · [C])<br>+(k3off · [CB2] · [B0])<br>- (k3on · [B2B0] · [C])<br>+(k3off · [CB0] · [B2])	-(k1off · [B2B0])<br>+(k1on · [B2] · [B0])<br>+(k5off · [CB2B1])<br>- (k5on · [B2B1] · [C])
d([B0B0)<br>dt	+(k1on · [B0] · [B0])<br>-(k1off · [B0B0])	+(k1on · [B0] · [B0])<br>-(k1off · [B0B0])<br>- (k3on · [B0B0] · [C])<br>+(k3off · [CB0] · [B0])	-(k1off · [B0B0])<br>+(k1on · [B0] · [B0])<br>+(k5off · [CB0B0])<br>- (k5on · [B0B0] · [C])
d([B1B2)<br>dt	+(k1on · [B1] · [B2])<br>-(k1off · [B1B2])	+(k1on · [B1] · [B2])<br>-(k1off · [B1B02)<br>- (k3on · [B1B2] · [C])<br>+(k3off · [CB1] · [B2])<br>- (k3on · [B1B2] · [C])<br>+(k3off · [CB2] · [B1])	-(k1off · [B1B2])<br>+(k1on · [B1] · [B2])<br>+(k5off · [CB1B2])<br>- (k5on · [B1B0] · [C])
d([CB0B0)<br>dt	-	-	-(k5off · [CB0B0])<br>+ (k5on · [B0B0] · [C])<br>- (k6on · [B0B0] · [C])<br>+(k6off · [CB0B0C])
d([CB1B1)<br>dt	-	-	-(k5off · [CB1B1])<br>+ (k5on · [B1B1] · [C])<br>- (k6on · [B1B1] · [C])<br>+(k6off · [CB1B1C])
d([CB2B2)<br>dt	-	-	-(k5off · [CB2B2])<br>+ (k5on · [B2B2] · [C])<br>- (k6on · [CB2B2] · [C])<br>+(k6off · [CB2B2C])
d([CB1B2)<br>dt	-	-	+(k5on · [B1B2])<br>- (k5off · [CB1B2])<br>- (k6on · [CB1B2] · [C])<br>+(k6off · [CB2B2C])
d([CB1B0)<br>dt	-	-	+(k5on · [B1B0])<br>- (k5off · [CB1B0])<br>- (k6on · [CB1B0] · [C])<br>+(k6off · [CB2B0C])

d([CB2B0)<br>dt	-	-	+(k5on · [B2B0] · [C])<br>- (k5off · [CB2B0])<br>- (k6on · [CB2B0] · [C])<br>+(k6off · [CB2B0C])
d([CB2B0C)<br>dt	-	-	- (k7off · [CB0B0C])<br>+(k7on · [CB0] · [CBO])<br>+ (k6on · [CB0B0] · [C])<br>-(k6off · [CB0B0C])
d([CB1B1C)<br>dt	-	-	- (k7off · [CB1B1C])<br>+(k7on · [CB1] · [CB1])<br>+ (k6on · [CB1B1] · [C])<br>-(k6off · [CB1B1C])
d([CB2B2C)<br>dt	-	-	- (k7off · [CB2B2C])<br>+(k7on · [CB2] · [CB2])<br>+ (k6on · [CB2B2] · [C])<br>-(k6off · [CB2B2C])
d([CB1B2C)<br>dt	-	-	- (k7off · [CB1B2C])<br>+(k7on · [CB1] · [CB2])<br>+ (k6on · [CB1B2] · [C])<br>-(k6off · [CB1B2C])
d([CB1B0C)<br>dt	-	-	- (k7off · [CB1B0C])<br>+(k7on · [CB1] · [CB0])<br>+ (k6on · [CB1B0] · [C])<br>-(k6off · [CB1B0C])
d([CB2B0C)<br>dt	-	-	- (k7off · [CB2B0C])<br>+(k7on · [CB2] · [CB0])<br>+ (k6on · [CB2B0] · [C])<br>-(k6off · [CB2B0C])

Supplementary Table 3: Ordinary differential equations of different model scenarios. B0 denotes unlabeled Bcl-xL, B1 denotes Bcl-xL_G, B2 denotes Bcl-xL_R and C denotes cBid. Concentrations are denoted by []. Kinetic rate constants are defined in Fig. 3A (k_+ denote k_{on} and k_- denote k_{off}).

Supplementary Methods

Fundamentals and technical details of fluorescence correlation spectroscopy (FCS) and Fluorescence cross correlation spectroscopy (FCCS)

This is a short summary on the method and the experimental setup used in this study. For more information about the method please read the following reviews [2-4].

FCCS measurements in solution

FCS measures the fluorescence fluctuations due to the diffusion of fluorophores or macromolecules (like proteins) with attached fluorophores through a small detection volume (<1fL), which is here created by a confocal microscope. The raw data of the experiment are fluorescence intensity traces and based on those auto-correlation curves are calculated. The auto-correlation curves contain information about the diffusion properties and the concentrations of the molecules studied. To gain quantitative information, mathematical fitting with appropriate models thus provides the number of molecules in the detection volume and their average diffusion time. The use of reference measurements with free fluorescent dyes of known diffusion coefficients allows calibration of the size and shape of the detection volume and estimation of fluorophore concentrations and diffusion coefficient. They are also useful to ensure the proper alignment of the instrument when are performed before each measurement. Here, we used the same dyes that are attached to the proteins of interest in the calibration measurements.

In FCS, the confocal microscope requires a high quality optical setup containing: objectives with a high numerical aperture, suitable laser lines, components to produce very well-shaped, small and maximum overlapping detection volumes, sensitive detectors (with nanosecond resolution), filters to avoid channel cross-talk, and the equipment to collect and analyze the FCCS data. In our case we used a LSM 710 with a Confocor 3 unit (Zeiss, Jena, Germany), optimized for FCS measurements. As the method has single molecule sensitivity, the best results are achieved when in the range of one molecule (per channel) is present in the detection volume. This is the case at nanomolar concentrations, so that we used mainly protein concentrations between 5-100 nM.

In FCCS (fluorescence cross correlation spectroscopy), the proteins of interest are labeled with spectrally different fluorophores that can be simultaneously excited (here by a 488 nm and a 633 nm laser). The emitted light from the two fluorophores is separated by optical parts (here dichroic mirrors and filters) and the resulting fluorescence intensity signals are acquired simultaneously with two detectors. Then, auto- and cross-correlation curves are calculated from the fluorescence intensity traces in both channels, which allow qualitative and quantitative assessment of the protein-protein-interactions.

The FCCS analysis of the data provides a percentage of cross-correlation (%CC) that can be used to calculate the extent of complex formation between the molecules of interest. For this, one needs to

correct for: i) partial overlap of the detection volumes of both channels; ii) degree of labeling of the proteins of interest is; and iii) fraction of free dye molecules in the sample.

Our experiments were optimized to detect Alexa488 or Atto488 in the "green" channel and Alexa633 or Atto655 in the "red" channel. Both "green" dyes were excited by the 488 nm laser and are detected using a 505-540 nm band pass filter and an Avalanche-Photo-Diode (APD) as detector. The "red" dyes were excited by the 633 nm laser and detected using a >655 nm long pass filter and a second APD.

The size of detection volume is directly proportional to the wavelength of the laser light. Thus, the detection volumes of both detection channels do not perfectly overlap, which precludes detecting 100% cross-correlation in both channels even when all molecules are fluorescently labeled and forming complexes. We performed test experiments to determine the maximal cross-correlation in each channel. An ideal sample to do so would be a stable molecule to which one red dye and one green dye is attached with 100% labeling efficiency, and no free dye present. Unfortunately, it was impossible for us produce or buy such a sample. Therefore, we used egg-PC vesicles (LUV) labeled with the two lipidic dyes: DID (exited by the 633 laser, emission maximum >655 nm) and DIO (excited by the 488 laser, emission maximum between 505-540 nm). Before the measurements, we passed the vesicle through a 200 nm filter to make them more homogenous in size. We detected >90% CC in the red channel and >75% CC in the green channel (Supplemental Fig. 1A), which provides an estimation of the partial overlap of both detection channels in our system. This is close to 100% cross-correlation in both channels, considering that the reference sample does not contain 100% doubly labeled particles with a zero background of free dye. With an ideal test sample, it would be possible to correct for incomplete spectral overlap by introducing a correction factor. Therefore, in our calculations we slightly underestimate the cross-correlation values, but as all measurements are affected in the same way, this only changes the absolute numbers and not our conclusions.

We chose the fluorophores based on: 1) a reasonable quantum yield, 2) good photo-stability, 3) their relative small size, 4) no detectable tendency to aggregate under the used condition (Supp. Fig. 1B), 5) that the diffusion of the fluorophore is well described in literature and 6) that the proteins of choice were still functional after fluorophore attachment. Alexa 488 and 633 were used to label cBid and Atto 488 and 655 to label Bax and Bcl-xL. None of those dyes showed signs of aggregation in their free form (example shown in Supplemental Fig. 1B) and the level of cross talk between the dye combinations used on the proteins was below 2% (Supp. Fig. 1C). An exception was the Alexa 488 /Alexa 633 pair (used only to study cBid homo-dimerization) with cross-talk levels up to 5%. However, as we did not detect any cBid homo-dimerization the higher cross-talk does not affect our conclusions. The free forms of the four dyes mentioned were also used to calculate the size and shape of the detection volumes.

For data quantification, the degree of labeling and the fraction of free dye in each protein sample were calculated. As shown in previous work, labeled cBid, Bax and Bcl-xL are active in membrane

permeabilization [1,5,6]. We also did visualize the protein before and after labeling by SDS-PAGE (Supplemental Fig. 1E). The degree of labeling was estimated using absorption spectra and mass spectroscopy. For Bax and Bcl-xL, we calculated degrees of labeling between 80 and 100%, while cBid was labeled between 60 to 80% (concrete info given in the figure legends).

Most fluorophore molecules were covalently attached to the proteins. However, the dyes can also bind non-covalently to protein surfaces. As free dye and "non-covalently protein attached" dye molecules are in equilibrium, the sample dilution before the FCS measurement will release some non-covalently attached dyes. This fraction of free dye needs to be considered for data quantification, which was done here by two component fitting of the auto-correlation curves. In the fitting process, the diffusion time of the free dye (known from reference measurements performed directly before the actual measurement) was fixed to reduce the free parameters, so that the fraction of labeled protein and free dye could be estimated with good accuracy (Supplemental Fig .1E). For Bax and Bcl-xL we calculated that the amount of free dye was ~20%, for cBid the amount was a bit higher (~ 20-30% for the green version and 30-40% for the red). The presence of non-covalently attached dyes is not unique to the Bcl-2 proteins. We have tested in our lab several other protein label combinations and after dilution to the low FCS concentration we were always able to detect some free dye molecules.

FCCS measurements in membranes

Due to the different viscosities of buffer solutions and lipid bilayers, membrane-embedded molecules diffuse slower than soluble ones, and they diffuse in two and not three dimensions. In addition, one important condition to measure FCS in membranes is to position the detection volume on the membrane plane. All this requires different measurement strategies, different fitting models and longer measurement times necessary. During the long measurement time, the sample can be bleached and the membrane may move in and out of the detection volume, which both difficult the analysis of the FCS data. Moreover, due to the differences in refraction index, the use of calibration measurements acquired in solution is problematic when quantitatively analyzing FCS data from membranes. To solve these problems, an FCS variant called two-focus scanning FCCS was developed for membrane proteins by the Schwille group and their work also provides the necessary mathematical models to fit the data [3,4].

In two-focus scanning FCCS, two parallel lines are repeatedly scanned perpendicular to and crossing the membrane of a GUV. As the laser is on the membrane only a fraction of the time, photobleaching is decreased. Moreover, the contributions of the membrane to the fluorescence intensity signal are aligned and integrated around the maximum, which allows corrections for slight movements of the membrane. If the distance between the two scanned lines is known, the data can be fitted with a mathematical model that provides directly diffusion coefficient and the structural parameter of the detection volume, so that calibration-free measurements are possible.

Key for two-focus scanning FCCS measurements are stable GUVs, in which the proteins of interest insert, interact and diffuse. Optimally, the membrane should mimic the physiological situation. In previous work, we investigated several suitable lipid mixtures to study Bcl-2 proteins in GUVs. Here we chose two different mixtures, which are described in the main text. Fortunately, the free form of the dyes used here do not interact with the GUV membranes [1,5,7], so that they do not need be considered for data quantification in FCS measurements in membranes.

Species used for model fitting to experimental data in different interaction scenarios:

Scenario 1 and Scenario 2:

[Bcl-x$L_{G\ particles}$] = [Bcl-xL_G] + [Bcl-xL_G-Bcl-xL_G] + [Bcl-xL_G-Bcl-xL_R] + [Bcl-xL_G-Bcl-xL_0] + [cBid-Bcl-xL_G]

[Bcl-x$L_{R\ particles}$] = [Bcl-xL_R] + [Bcl-xL_R-Bcl-xL_R] + [Bcl-xL_G-Bcl-xL_R] + [Bcl-xL_R-Bcl-xL_0] + [cBid-Bcl-xL_R]

[Bcl-xL_{G-} Bcl-x$L_{R\ particles}$] =[Bcl-xL_{G-} Bcl-xL_R]

Scenario 3

[Bcl-x$L_{G\ particles}$] = [Bcl-xL_G] + [Bcl-xL_G-Bcl-xL_G] + [Bcl-xL_G-Bcl-xL_R] + [Bcl-xL_G-Bcl-xL_0] + [cBid-Bcl-xL_G] + [cBid-Bcl-xL_G-Bcl-xL_G] + [cBid-Bcl-xL_G-Bcl-xL_R] + [cBid-Bcl-xL_G-Bcl-xL_o] + [cBid- Bcl-xL_{G-} Bcl-xL_G-cBid] + [cBid- Bcl-xL_{G-} Bcl-xL_R-cBid] + [cBid- Bcl-xL_{G-} Bcl-xL_0-cBid]

[Bcl-x$L_{R\ particles}$] = [Bcl-xL_R] + [Bcl-xL_R-Bcl-xL_R] + [Bcl-xL_G-Bcl-xL_R] + [Bcl-xL_R-Bcl-xL_0] + [cBid-Bcl-xL_R] +[cBid-Bcl-xL_R-Bcl-xL_R] + [cBid-Bcl-xL_G-Bcl-xL_R] + [cBid-Bcl-xL_R-Bcl-xL_o] + [cBid- Bcl-xL_{R-} Bcl-xL_R-cBid] + [cBid- Bcl-xL_{G-} Bcl-xL_R-cBid] + [cBid- Bcl-xL_{R-} Bcl-xL_0-cBid]

[Bcl-xL_G - Bcl-x$L_{R\ particles}$] =[Bcl-xL_{G-} Bcl-xL_R]+[cBid- Bcl-xL_{G-} Bcl-xL_R] + [cBid- Bcl-xL_{G-} Bcl-xL_R-cBid]

Calculation of Bcl-xL dimer and monomer concentrations from FCS measurements for plot in Suppl. Fig. 1 was performed the following way, assuming equilibrium conditions:

$DOL_{B\bullet}$ labelling effeciency of Bcl-xL_G (B•)

$DOL_{B\circ}$ labelling effeciency of Bcl-xL_R (B∘)

measured values from FCS:

[B•-B∘]

$[B\bullet]_{tot}$ = [B•] + [B•-B•] + [B•-B∘] + [B•-B]

$[B\circ]_{tot}$ = [B∘] + [B∘-B∘] + [B•-B∘] + [B∘-B]

values in plot:

$[B]_{tot,free} = [B\bullet] + [B\circ] + [B]$

$[B\text{-}B]_{tot} = [B\bullet\text{-}B\bullet] + [B\bullet\text{-}B\circ] + [B\circ\text{-}B\circ] + [B\bullet\text{-}B] + [B\circ\text{-}B] + [B\text{-}B]$

calculation of [B∘](9), [B•](8), [B](7), [B•-B•](2), [B∘-B∘](3), [B-B](4), [B•-B](5) and [B∘-B](6) :

(1) $$K_D = \frac{[B\bullet]^2}{[B\bullet - B\bullet]} = \frac{[B\circ]^2}{[B\circ - B\circ]} = \frac{[B]^2}{[B-B]} = \frac{[B\bullet]\cdot[B\circ]}{[B\bullet - B\circ]} = \frac{[B\bullet]\cdot[B]}{[B\bullet - B]} = \frac{[B\circ]\cdot[B]}{[B\circ - B]}$$

(2)-(6) follow from (1):

(2) $$[B\bullet - B\bullet] = \frac{[B\bullet]\cdot[B\bullet - B\circ]}{[B\circ]}$$

(3) $$[B\circ - B\circ] = \frac{[B\circ]\cdot[B\bullet - B\circ]}{[B\bullet]}$$

(4) $$[B - B] = \frac{[B]^2\cdot[B\bullet - B\circ]}{[B\bullet]\cdot[B\circ]}$$

(5) $$[B\bullet - B] = \frac{[B]\cdot[B\bullet - B\circ]}{[B\circ]}$$

(6) $$[B\circ - B] = \frac{[B]\cdot[B\bullet - B\circ]}{[B\bullet]}$$

(7) $$[B] = [B\circ]\cdot\left(\frac{1}{DOL_{B\circ}} - 1\right) + [B\bullet]\cdot\left(\frac{1}{DOL_{B\bullet}} - 1\right)$$

from (1):

$$[B\bullet]_{tot} = [B\bullet] + \frac{[B\bullet]^2}{K_D} + \frac{[B\bullet]\cdot[B\circ]}{K_D} + \frac{[B\bullet]\cdot[B]}{K_D}$$

$$[B\bullet]_{tot} = [B\bullet]\cdot\left(1 + \frac{[B\bullet]}{K_D} + \frac{[B\circ]}{K_D} + \frac{[B]}{K_D}\right)$$

$$[B\circ]_{tot} = [B\circ]\cdot\left(1 + \frac{[B\circ]}{K_D} + \frac{[B\bullet]}{K_D} + \frac{[B]}{K_D}\right)$$

(8) $$[B\bullet] = \frac{[B\bullet]_{tot}\cdot[B\circ]}{[B\circ]_{tot}}$$

Rearranging $[B\circ]_{tot}$ leads to

$$[B\circ] = [B\circ]_{tot} - [B\circ - B\circ] - [B\bullet - B\circ] - [B\circ - B]$$

(9) follows then from (3), (6), (7) and (8):

$$[B\circ] = [B\circ]_{tot} - \frac{[B\bullet - B\circ]\cdot[B\circ]_{tot}}{[B\bullet]_{tot}} - [B\bullet - B\circ] - \frac{[B\circ]_{tot}\cdot[B\bullet - B\circ]\cdot\left(\frac{1}{DOL_{B\circ}} - 1 + \frac{[B\bullet]_{tot}}{DOL_{B\bullet}\cdot[B\circ]_{tot}} - \frac{[B\bullet]_{tot}}{[B\circ]_{tot}}\right)}{[B\bullet]_{tot}}$$

(1) K_D was then be determined by plotting $[B]_{tot,free}^2$ against $[B\text{-}B]_{tot}$ and determining the slope of the linear regression line.

Supplementary References:

1 Bleicken, S., Wagner, C. & García-Sáez, Ana J. Mechanistic Differences in the Membrane Activity of Bax and Bcl-xL Correlate with Their Opposing Roles in Apoptosis. *Biophysical Journal* **104**, 421-431, (2013).

2 Ries, J., Petrasek, Z., Garcia-Saez, A. J. & Schwille, P. A comprehensive framework for fluorescence cross-correlation spectroscopy. *New J. Phys.* **12**, (2010).

3 Ries, J. & Schwille, P. New concepts for fluorescence correlation spectroscopy on membranes. *Phys Chem Chem Phys* **10**, 3487-3497, (2008).

4 Ries, J., Weidemann, T. & Schwille, P. in *Comprehensive Biophysics* (ed Edward H. Egelman) 210-245 (Elsevier, 2012).

5 Bleicken, S., Landeta, O., Landajuela, A., Basanez, G. & Garcia-Saez, A. J. Proapoptotic Bax and Bak form stable protein-permeable pores of tunable size. *J Biol Chem* **288**, 33241-33252, (2013).

6 Subburaj, Y. *et al.* Bax monomers form dimer units in the membrane that further self-assemble into multiple oligomeric species. *Nat Commun* **6**, (2015).

7 Bleicken, S. *et al.* Structural Model of Active Bax at the Membrane. *Molecular Cell* **56**, 496-505, (2014).
